# Supplementary material for: The representation of context in mouse hippocampus is preserved despite neural drift
Source: Nat Commun. 2022 May 3;13:2415. doi: 10.1038/s41467-022-30198-7 (PMC9065029; doi:10.1038/s41467-022-30198-7)
Supplement: Supplementary file 1 — Supplementary Information [file 41467_2022_30198_MOESM1_ESM.pdf]

Supplementary information for:

The representation of context in mouse hippocampus is preserved despite neural drift

Keinath, et al.

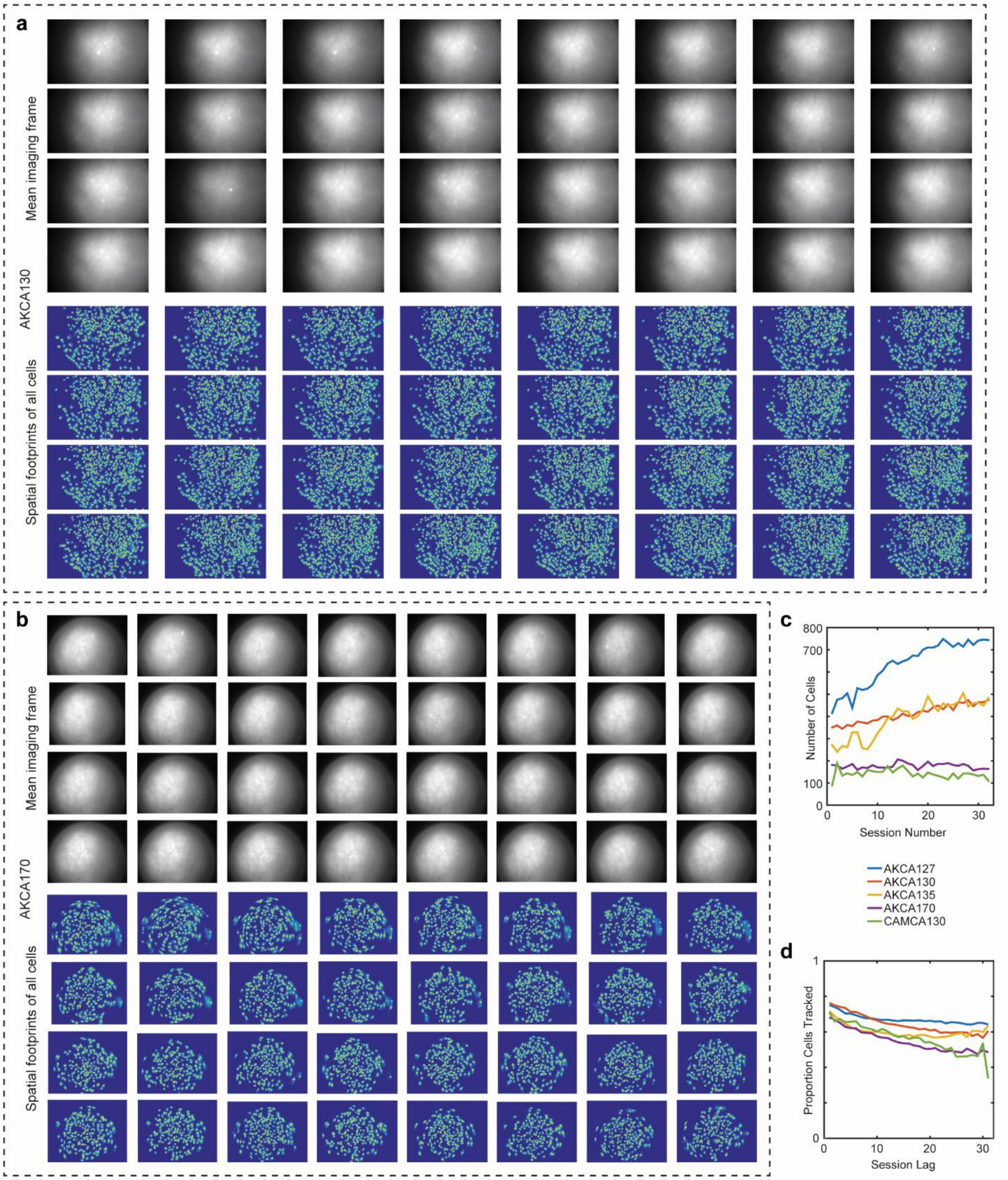

Supplementary Figure 1. Imaging across 32 days of recording. Two example mice, AKCA130 (**a**) implanted with a 1.8 mm lens and AKCA170 (**b**) implanted with a 0.5 mm lens. Mean imaging frame normalized to maximum. Spatial footprints scaled and thresholded for ease of interpretation. Arranged chronologically. (**c**) Cell counts for each mouse across all 32 days. (**d**) Proportion of the population registered as a function of lag between sessions for each mouse. Normalized to the smaller population for each pairwise comparison.

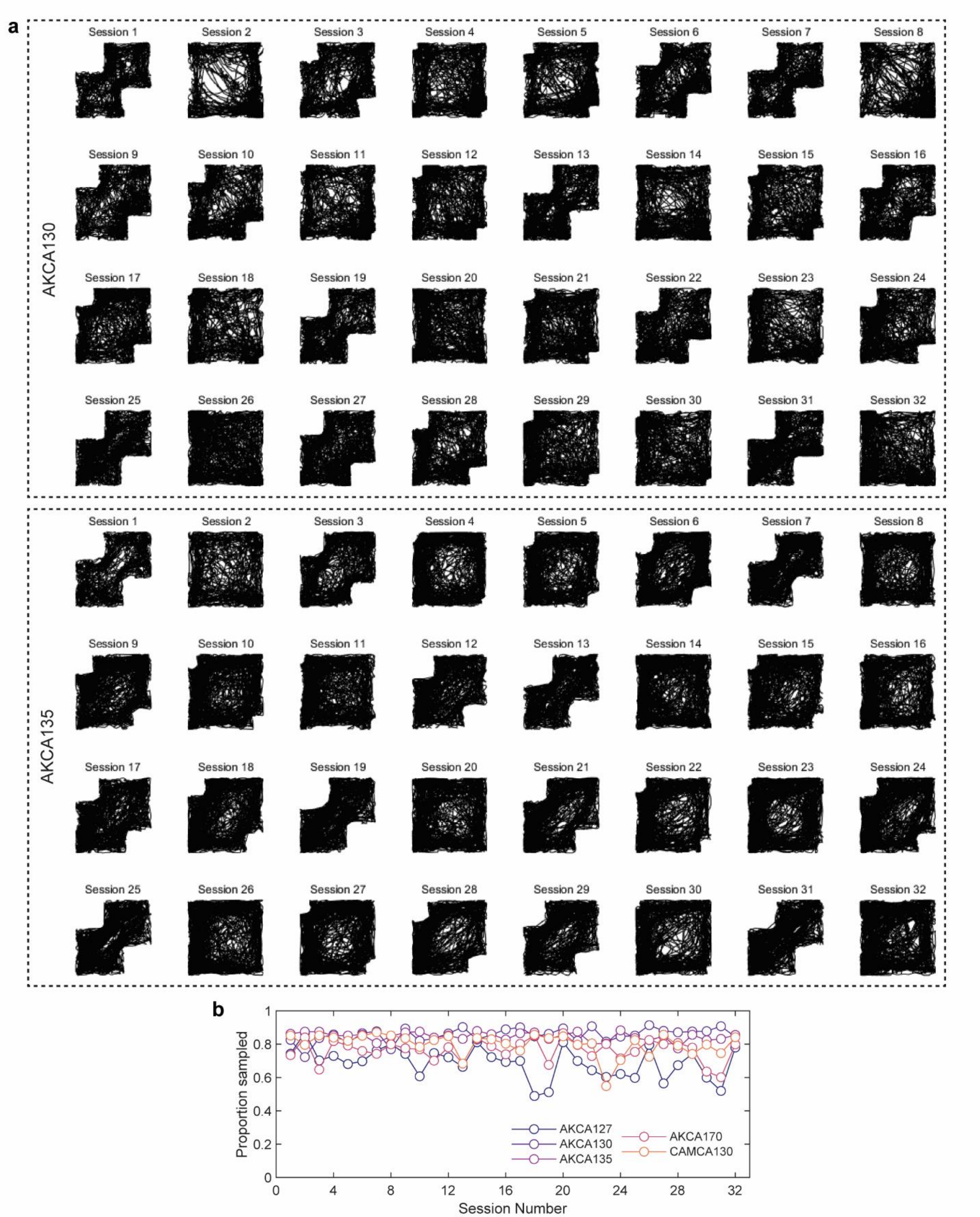

Supplementary Figure 2. Spatial sampling across all 32 sessions. (**a**) Tracked position for two example mice. (**b**) Proportion of the environment sampled for all sessions and all mice. Computed as the proportion of 2.5 cm x 2.5 cm bins visited for at least 1 s. For reference, perfectly even sampling in the square environment would yield 4.69 s of sampling per bin.

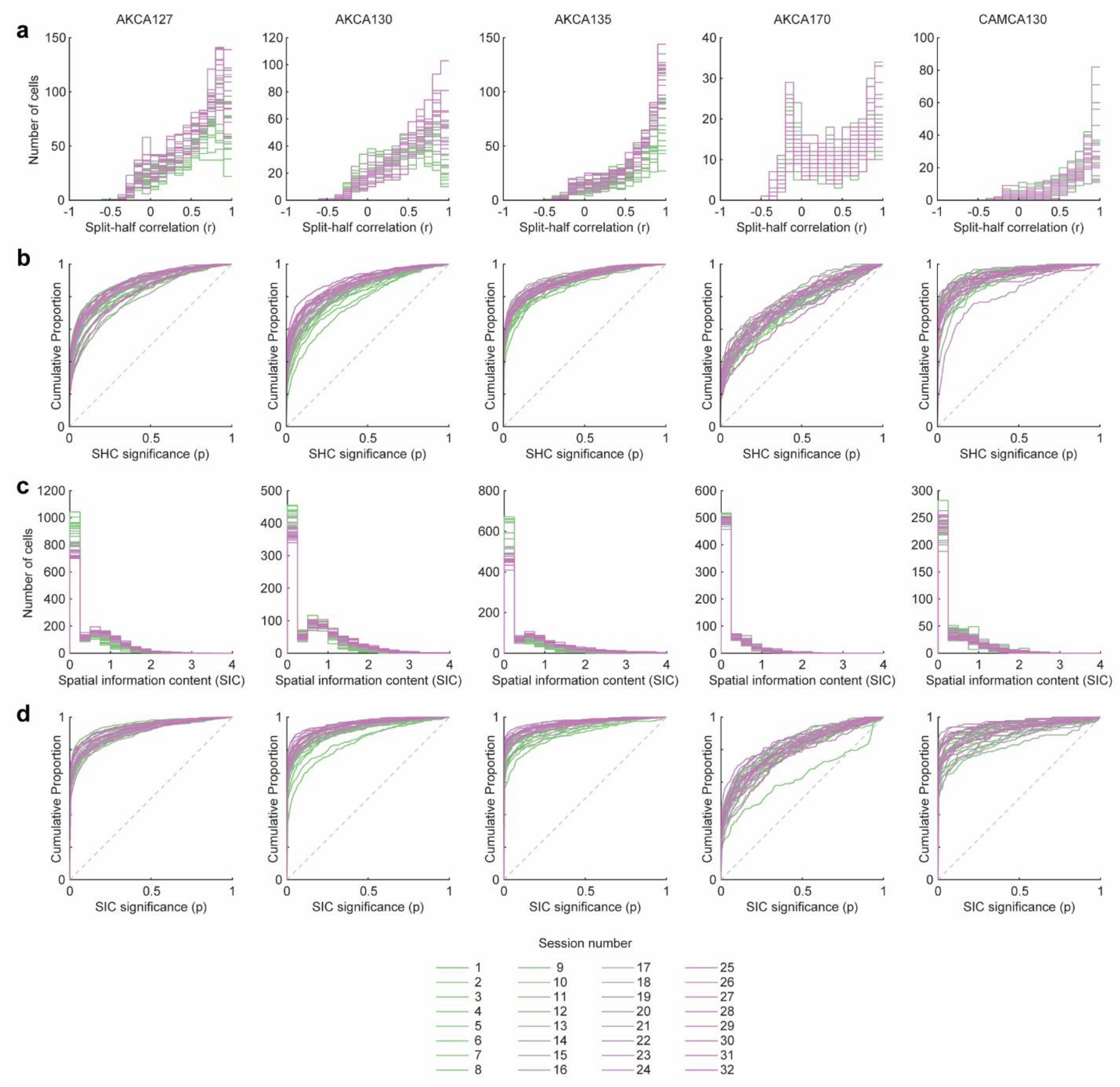

Supplementary Figure 3. Measures of place code quality for all sessions and mice. (**a**) Raw split-half Pearson's rate map correlation values (SHC). (**b**) SHC significance relative to a shuffled distribution for each cell (1000 random circular shifts of the trace data relative to the position data at least 60 s away from its proper alignment). (**c**) Spatial information content (SIC). (**d**) SIC significance relative to a shuffled distribution for each cell (1000 random circular shifts of the trace data relative to the position data at least 60 s away from its proper alignment).

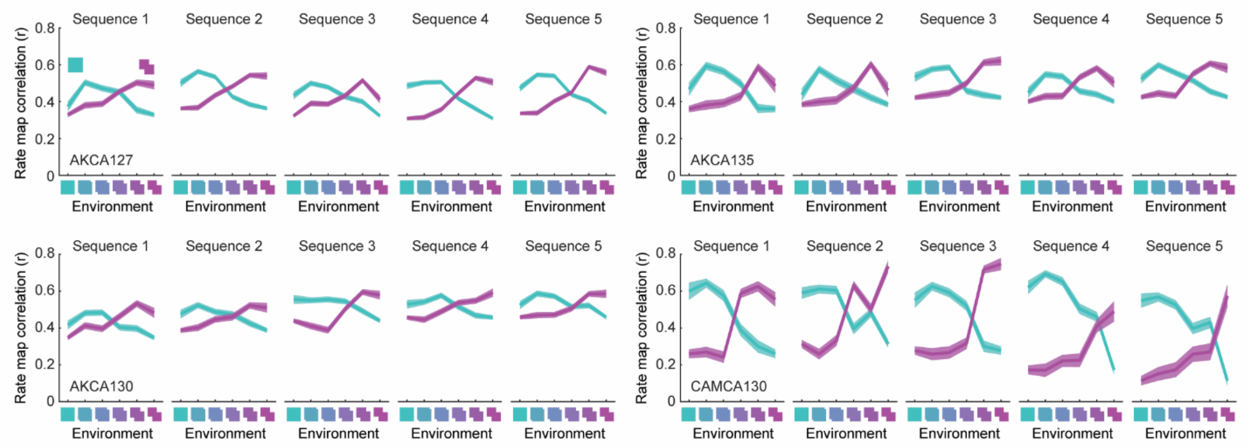

Supplementary Figure 4. Within-sequence transition plots for remaining mice and sessions. *Rate map correlations between each environment and the two familiar environments for all five eight-day bookended morph sequences for the remaining four mice. Mouse ID in lower left. Lines and shading denote mean ± 1 SEM across all tracked cells whose within-session split-half reliability exceeding the 95*th *percentile of a shuffled distribution for at least one of the compared sessions.*

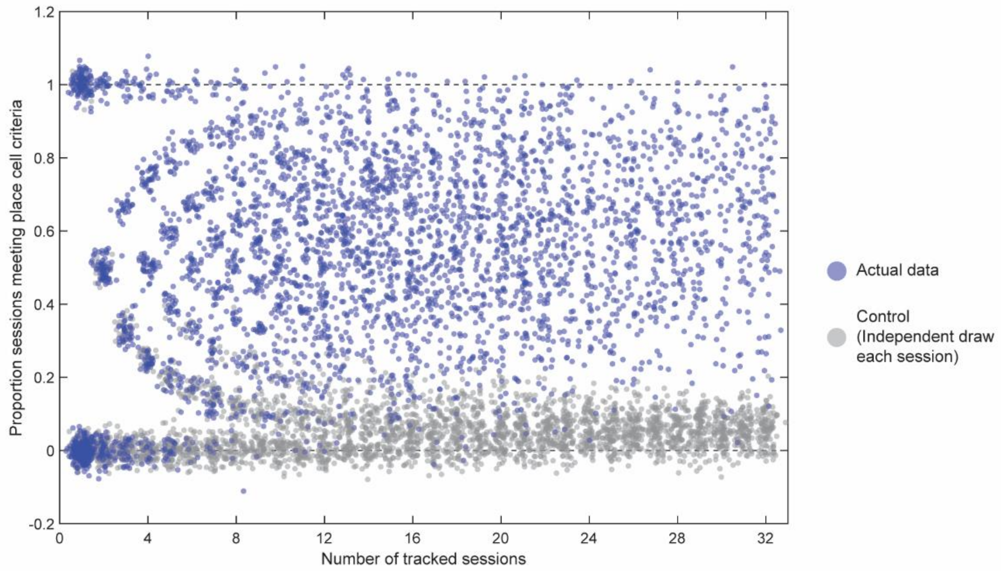

Supplementary Figure 5. Proportion of sessions meeting place cell criteria for each cell as a function of number of registered sessions. A cell was defined as meeting place cell criteria within a given session if its split-half rate map correlation value exceeded the 95th percentile of its shuffled distribution (1000 random circular shifts of the trace data relative to the position data at least 60 s away from its proper alignment). For comparison, a control of independent draws at a chance level of 0.05 for from each session is also plotted. A small amount of mean-zero Gaussian noise (x: standard deviation = 0.25; y: standard deviation = 0.025) was added to each point to increase interpretability. Note that the proportion of sessions for which a given cell meets place cell criteria is rarely bimodal when a sufficient number of sessions is sampled, indicating that a failure to meet place cell criteria on a given day does not imply that the same cell will fail to meet criteria on future sessions.

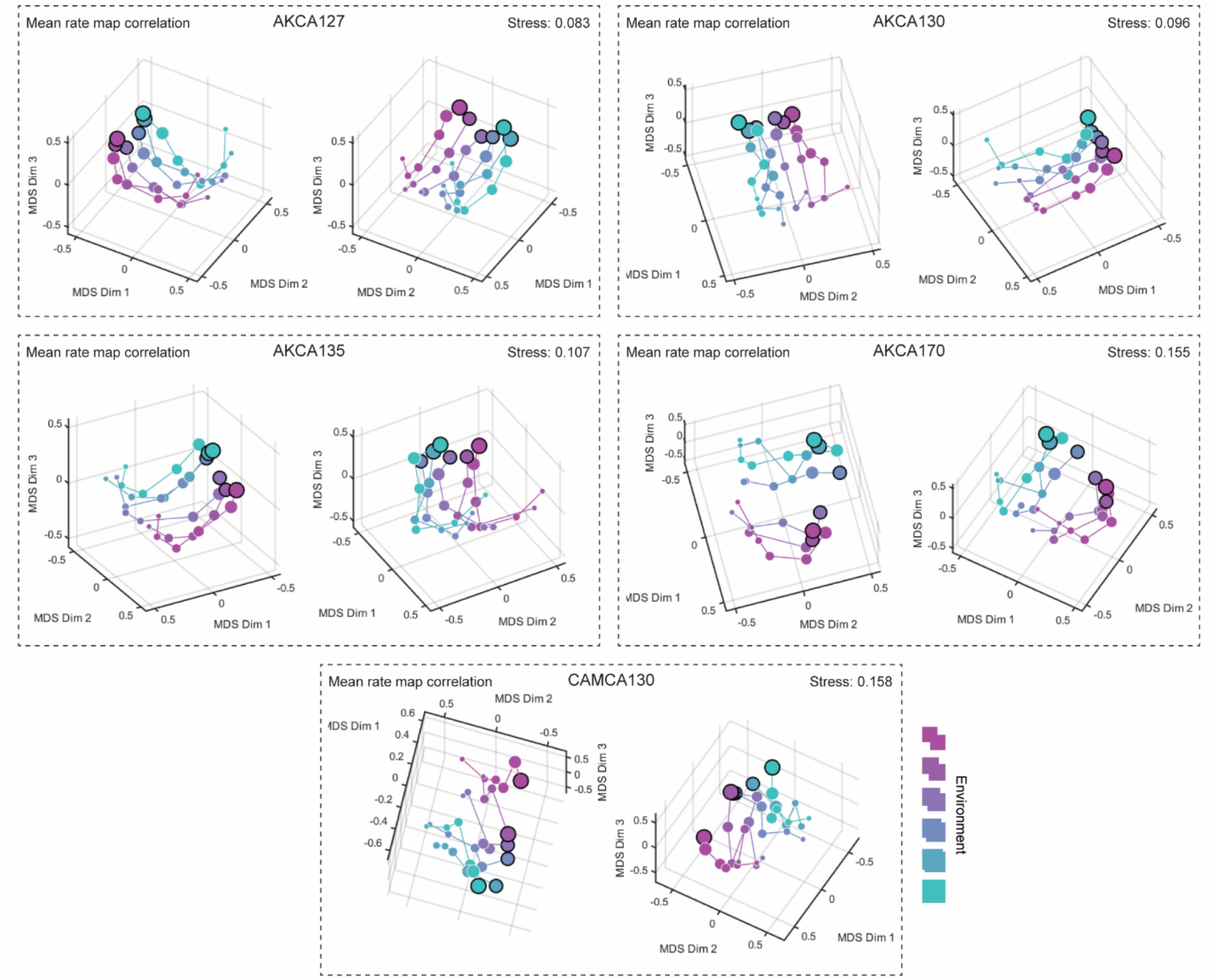

Supplementary Figure 6. nMDS results for three dimensions with mean rate map correlation as the measure of similarity between sessions. Note that, as in two-dimensions, the embedded dimension distinguishing context is generally orthogonal to the dimensions distinguishing long-timescale changes.

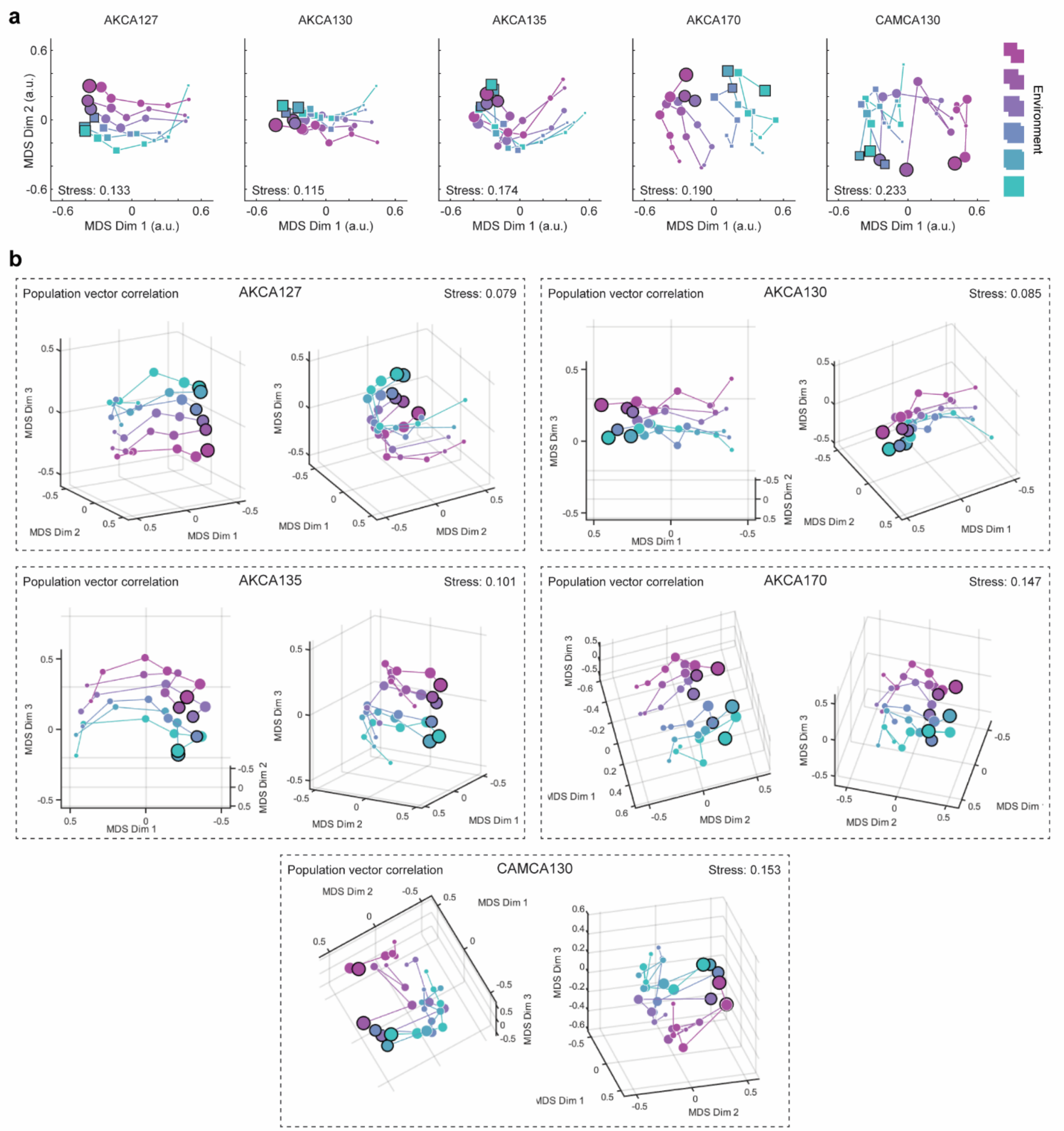

Supplementary Figure 7. nMDS results with population vector correlation as the measure of similarity between sessions. (**a**) Two-dimensional embeddings. (**b**) three-dimensional embeddings. Note that stress values are higher for two-dimensional embeddings, indicating that three-dimensional embeddings are more appropriate for interpretation. Also note that, as with taking mean rate map correlation as the measure of similarity between sessions, the embedded dimension distinguishing context is generally orthogonal to the dimensions distinguishing long-timescale changes. a.u. denotes arbitrary units.

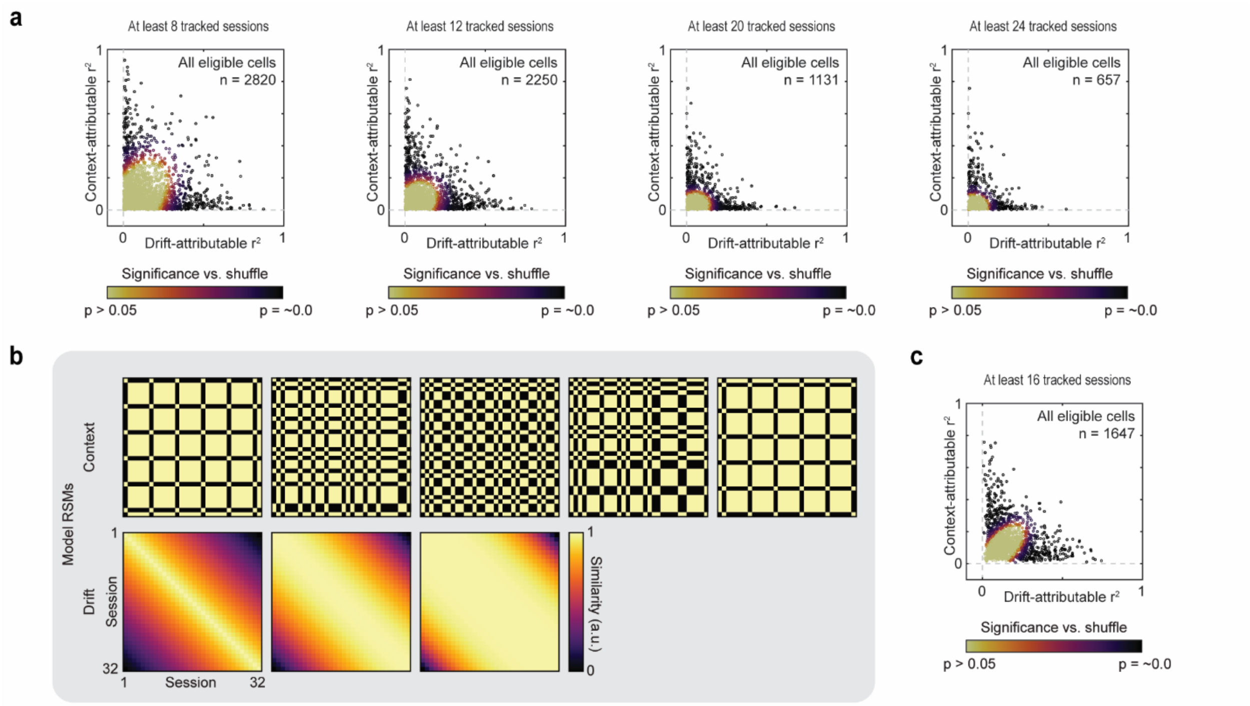

Supplementary Figure 8. Similar heterogeneity of attributable explained variance for individual cell RSMs is observed when varying inclusion criteria and model RSMs. (**a**) Distribution of explained variance attributable to drift and contextual factors for all cells identified on a variety of minimum number of sessions, combined across animals. Color indicates significance relative to a shuffled control as described in the main text. (**b**) Schematic of an alternative model. Drift RSMs were specified a linear, quadratic, and fourth-power decrease in similarity as a function of the number of days between each pairwise session comparison. Contextual RSMs specified high similarity between sessions from the same side of the shapespace and low similarity otherwise, with a separate RSM defining the side of the shape space as occurring between each pair of environments which neighbor one another in shape space (i.e. if environments laid out in shapespace order and are labeled 1 through 6, one contextual RSM sets the cut-off point for same side of the shapespace as occurring between environments 1 and 2, another RSM between 2 and 3, another RSM between 3 and 4, and so forth). To constrain the model to a reasonable number of terms, only second order interactions across pairs of drift and contextual RSMs were allowed, resulting in 24 total terms (3 drift RSMs + 5 Context RSMs + 15 Drift*Context RSM interaction terms + a constant term) in the full model. (**c**) Distribution of explained variance attributable to drift and contextual factors for all cells identified on at least 16 sessions, combined across animals, when applying the model specified in (**b**). Color indicates significance relative to a shuffled control as described in the main text.

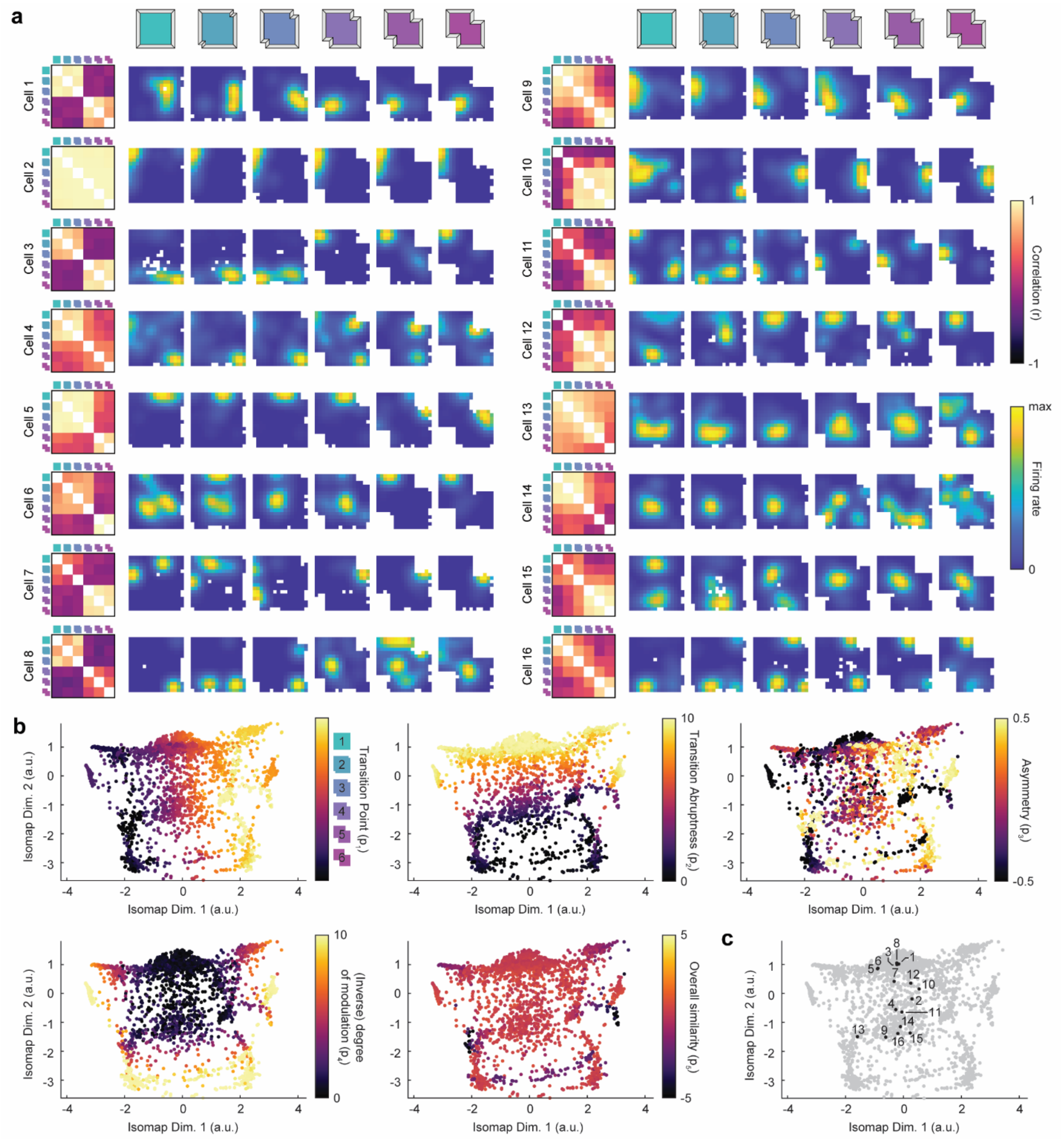

Supplementary Figure 9. Heterogeneity of individual cell contextual RSMs. (**a**) Sixteen examples of individual cell contextual RSMs and the 6-day morph sequence rate maps from which these RSMs were generated. (**b**) To visualize the heterogeneity of contextual RSMs, we embedded each RSM in a two-dimensional space by first fitting each RSM with a five-parameter sigmoidal model (see Methods for details including parameter descriptions). Next we reduced this five-parameter fit to two dimensions via Isomap[1], a nonlinear dimensionality reduction technique which builds on MDS to preserve local relationships between neighboring datapoints (k = 100 nearest neighbors; Mahalanobis distance between fit parameters). Finally, we color-coded the embedded RSMs according to each of the five fit parameters, to provide a sense for how this embedding relates to each aspect of the sigmoidal model. (**c**) The same embedding as in (**b**) except with the locations of our 16 example RSMs from (**a**) indicated. a.u. denotes arbitrary units.

Inclusion	Measure	Conditions	Statistical test	Outcome
All identified cells	Context group	Lag 1-6	t-test vs 0.5 (1-tailed)	t(4) = 10.54, p = 2.29e-04
	Context group	Lag 7-12	t-test vs 0.5 (1-tailed)	t(4) = 9.35, p = 3.65e-04
	Context group	Lag 13-18	t-test vs 0.5 (1-tailed)	t(4) = 3.09, p = 1.83e-02
	Context group	Lag 19+	t-test vs 0.5 (1-tailed)	t(4) = 3.47, p = 1.27e-02
	Context group	Between All	ANOVA	F(3,16) = 0.53, p = 6.68e-01
	Environment ID	Lag 1-6	t-test vs 0.5 (1-tailed)	t(4) = 3.93, p = 8.53e-03
	Environment ID	Lag 7-12	t-test vs 0.5 (1-tailed)	t(4) = 5.43, p = 2.78e-03
	Environment ID	Lag 13-18	t-test vs 0.5 (1-tailed)	t(4) = 2.31, p = 4.10e-02
	Environment ID	Lag 19+	t-test vs 0.5 (1-tailed)	t(4) = 1.87, p = 6.75e-02
	Environment ID	Between All	ANOVA	F(3,16) = 0.01, p = 9.99e-01
	Best Match *r*	Between All	ANOVA	F(3,16) = 14.99, p = 6.58e-05
	Best Match *r*	Lag 1-6 vs 7-12	paired t-test (2-tailed)	t(4) = 5.02, p = 7.40e-03
	Best Match *r*	Lag 1-6 vs 13-18	paired t-test (2-tailed)	t(4) = 6.05, p = 3.76e-03
	Best Match *r*	Lag 1-6 vs 19+	paired t-test (2-tailed)	t(4) = 7.30, p = 1.87e-03
	Best Match *r*	Lag 7-12 vs 13-18	paired t-test (2-tailed)	t(4) = 7.80, p = 1.46e-03
	Best Match *r*	Lag 7-12 vs 19+	paired t-test (2-tailed)	t(4) = 9.74, p = 6.22e-04
	Best Match *r*	Lag 13-18 vs 19+	paired t-test (2-tailed)	t(4) = 8.98, p = 8.50e-04
All cells, substitute zeros for missing cell rate maps	Context group	Lag 1-6	t-test vs 0.5 (1-tailed)	t(4) = 4.07, p = 7.61e-03
	Context group	Lag 7-12	t-test vs 0.5 (1-tailed)	t(4) = 4.09, p = 7.52e-03
	Context group	Lag 13-18	t-test vs 0.5 (1-tailed)	t(4) = 3.50, p = 1.25e-02
	Context group	Lag 19+	t-test vs 0.5 (1-tailed)	t(4) = 3.21, p = 1.63e-02
	Context group	Between All	ANOVA	F(3,16) = 0.31, p = 8.21e-01
	Environment ID	Lag 1-6	t-test vs 0.5 (1-tailed)	t(4) = 3.45, p = 1.30e-02
	Environment ID	Lag 7-12	t-test vs 0.5 (1-tailed)	t(4) = 2.67, p = 2.80e-02
	Environment ID	Lag 13-18	t-test vs 0.5 (1-tailed)	t(4) = 2.36, p = 3.90e-02
	Environment ID	Lag 19+	t-test vs 0.5 (1-tailed)	t(4) = 1.71, p = 8.14e-02
	Environment ID	Between	ANOVA	F(3,16) = 0.49, p = 6.96e-01
	Best Match *r*	Between All	ANOVA	F(3,16) = 23.71, p = 3.95e-06
	Best Match *r*	Lag 1-6 vs 7-12	paired t-test (2-tailed)	t(4) = 8.32, p = 1.14e-03
	Best Match *r*	Lag 1-6 vs 13-18	paired t-test (2-tailed)	t(4) = 9.39, p = 7.17e-04
	Best Match *r*	Lag 1-6 vs 19+	paired t-test (2-tailed)	t(4) = 10.85, p = 4.09e-04
	Best Match *r*	Lag 7-12 vs 13-18	paired t-test (2-tailed)	t(4) = 10.63, p = 4.44e-04
	Best Match *r*	Lag 7-12 vs 19+	paired t-test (2-tailed)	t(4) = 10.65, p = 4.41e-04
	Best Match *r*	Lag 13-18 vs 19+	paired t-test (2-tailed)	t(4) = 7.02, p = 2.17e-03

Supplementary Table 1. Outcomes from statistical tests for prediction of contextual information. All p-values are uncorrected.

Lag	Test	Comparisons	Statistical outcome	Lag	Test	Comparisons	Statistical outcome
1	ANOVA	all	F(5,1494) = 23.65, p = 6.02e-23	3	ANOVA	all	F(5,1410) = 23.71, p = 6.05e-23
1	t-test	1 vs. 2	t(498) = 2.98, p = 3.01e-03	3	t-test	1 vs. 2	t(470) = 3.54, p = 4.46e-04
1	t-test	1 vs. 3	t(498) = 5.20, p = 2.90e-07	3	t-test	1 vs. 3	t(470) = 5.57, p = 4.36e-08
1	t-test	1 vs. 4	t(498) = 7.20, p = 2.24e-12	3	t-test	1 vs. 4	t(470) = 7.58, p = 1.82e-13
1	t-test	1 vs. 5	t(498) = 7.69, p = 7.82e-14	3	t-test	1 vs. 5	t(470) = 8.18, p = 2.68e-15
1	t-test	1 vs. 6	t(498) = 8.41, p = 4.19e-16	3	t-test	1 vs. 6	t(470) = 8.60, p = 1.24e-16
1	t-test	2 vs. 3	t(498) = 2.44, p = 1.49e-02	3	t-test	2 vs. 3	t(470) = 2.02, p = 4.40e-02
1	t-test	2 vs. 4	t(498) = 4.60, p = 5.47e-06	3	t-test	2 vs. 4	t(470) = 3.97, p = 8.15e-05
1	t-test	2 vs. 5	t(498) = 5.17, p = 3.32e-07	3	t-test	2 vs. 5	t(470) = 4.63, p = 4.74e-06
1	t-test	2 vs. 6	t(498) = 5.97, p = 4.48e-09	3	t-test	2 vs. 6	t(470) = 5.13, p = 4.19e-07
1	t-test	3 vs. 4	t(498) = 2.10, p = 3.62e-02	3	t-test	3 vs. 4	t(470) = 1.92, p = 5.49e-02
1	t-test	3 vs. 5	t(498) = 2.73, p = 6.63e-03	3	t-test	3 vs. 5	t(470) = 2.62, p = 9.05e-03
1	t-test	3 vs. 6	t(498) = 3.53, p = 4.57e-04	3	t-test	3 vs. 6	t(470) = 3.18, p = 1.56e-03
1	t-test	4 vs. 5	t(498) = 0.68, p = 4.97e-01	3	t-test	4 vs. 5	t(470) = 0.75, p = 4.51e-01
1	t-test	4 vs. 6	t(498) = 1.50, p = 1.34e-01	3	t-test	4 vs. 6	t(470) = 1.39, p = 1.66e-01
1	t-test	5 vs. 6	t(498) = 0.81, p = 4.19e-01	3	t-test	5 vs. 6	t(470) = 0.65, p = 5.18e-01
2	ANOVA	all	F(5,1548) = 38.63, p = 1.81e-37	4	ANOVA	all	F(5,768) = 11.52, p = 9.54e-11
2	t-test	1 vs. 2	t(516) = 3.30, p = 1.03e-03	4	t-test	1 vs. 2	t(256) = 0.99, p = 3.21e-01
2	t-test	1 vs. 3	t(516) = 7.19, p = 2.25e-12	4	t-test	1 vs. 3	t(256) = 3.86, p = 1.45e-04
2	t-test	1 vs. 4	t(516) = 9.18, p = 1.03e-18	4	t-test	1 vs. 4	t(256) = 4.60, p = 6.67e-06
2	t-test	1 vs. 5	t(516) = 8.79, p = 2.18e-17	4	t-test	1 vs. 5	t(256) = 4.40, p = 1.61e-05
2	t-test	1 vs. 6	t(516) = 11.56, p = 1.16e-27	4	t-test	1 vs. 6	t(256) = 5.51, p = 8.79e-08
2	t-test	2 vs. 3	t(516) = 4.21, p = 3.01e-05	4	t-test	2 vs. 3	t(256) = 3.21, p = 1.50e-03
2	t-test	2 vs. 4	t(516) = 6.34, p = 4.97e-10	4	t-test	2 vs. 4	t(256) = 4.05, p = 6.76e-05
2	t-test	2 vs. 5	t(516) = 5.91, p = 6.40e-09	4	t-test	2 vs. 5	t(256) = 3.82, p = 1.65e-04
2	t-test	2 vs. 6	t(516) = 8.79, p = 2.26e-17	4	t-test	2 vs. 6	t(256) = 5.08, p = 7.17e-07
2	t-test	3 vs. 4	t(516) = 2.11, p = 3.55e-02	4	t-test	3 vs. 4	t(256) = 0.93, p = 3.55e-01
2	t-test	3 vs. 5	t(516) = 1.64, p = 1.02e-01	4	t-test	3 vs. 5	t(256) = 0.70, p = 4.87e-01
2	t-test	3 vs. 6	t(516) = 4.29, p = 2.12e-05	4	t-test	3 vs. 6	t(256) = 1.95, p = 5.26e-02
2	t-test	4 vs. 5	t(516) = -0.49, p = 6.25e-01	4	t-test	4 vs. 5	t(256) = -0.22, p = 8.23e-01
2	t-test	4 vs. 6	t(516) = 2.07, p = 3.90e-02	4	t-test	4 vs. 6	t(256) = 0.99, p = 3.24e-01
2	t-test	5 vs. 6	t(516) = 2.60, p = 9.45e-03	4	t-test	5 vs. 6	t(256) = 1.21, p = 2.26e-01

Supplementary Table 2. Outcomes from statistical tests for stability of cells grouped by contextual RSM fit after matching for mean firing rate. All p-values are uncorrected. All t-tests are two-sample and two-tailed.

Supplementary References

1. Tenenbaum, J. B., de Silva, V. & Langford, J. C. A global geometric framework for nonlinear dimensionality reduction. *Science* **290**, 2319–23 (2000).
