# Supplementary material for: The representation of context in mouse hippocampus is preserved despite neural drift
Source: Nat Commun. 2022 May 3;13:2415. doi: 10.1038/s41467-022-30198-7 (PMC9065029; doi:10.1038/s41467-022-30198-7)
Supplement: Supplementary file 4 — Reporting Summary [file 41467_2022_30198_MOESM4_ESM.pdf]

nature research

Corresponding author(s): Keinath and Brandon

Last updated by author(s): Mar 15, 2022

Reporting Summary

Nature Research wishes to improve the reproducibility of the work that we publish. This form provides structure for consistency and transparency in reporting. For further information on Nature Research policies, see our Editorial Policies and the Editorial Policy Checklist.

Statistics

For all statistical analyses, confirm that the following items are present in the figure legend, table legend, main text, or Methods section.

n/a	Confirmed	
☐	☒	The exact sample size (*n*) for each experimental group/condition, given as a discrete number and unit of measurement
☐	☒	A statement on whether measurements were taken from distinct samples or whether the same sample was measured repeatedly
☐	☒	The statistical test(s) used AND whether they are one- or two-sided<br>*Only common tests should be described solely by name; describe more complex techniques in the Methods section.*
☐	☒	A description of all covariates tested
☒	☐	A description of any assumptions or corrections, such as tests of normality and adjustment for multiple comparisons
☐	☒	A full description of the statistical parameters including central tendency (e.g. means) or other basic estimates (e.g. regression coefficient) AND variation (e.g. standard deviation) or associated estimates of uncertainty (e.g. confidence intervals)
☐	☒	For null hypothesis testing, the test statistic (e.g. *F*, *t*, *r*) with confidence intervals, effect sizes, degrees of freedom and *P* value noted<br>*Give P values as exact values whenever suitable.*
☒	☐	For Bayesian analysis, information on the choice of priors and Markov chain Monte Carlo settings
☒	☐	For hierarchical and complex designs, identification of the appropriate level for tests and full reporting of outcomes
☐	☒	Estimates of effect sizes (e.g. Cohen's *d*, Pearson's *r*), indicating how they were calculated

Our web collection on statistics for biologists contains articles on many of the points above.

Software and code

Policy information about availability of computer code

Data collection	In vivo calcium videos were recorded with a UCLA miniscope (v3; miniscope.org) containing a monochrome CMOS imaging sensor (MT9V032C12STM, ON Semiconductor) connected to a custom data acquisition (DAQ) box (miniscope.org) with a lightweight, flexible coaxial cable. The DAQ was connected to a PC with a USB 3.0 SuperSpeed cable and controlled with Miniscope custom acquisition software (miniscope.org; https://github.com/daharoni/Miniscope_DAQ_Software/tree/master/x64/Release).
Data analysis	Calcium imaging data were preprocessed prior to analyses via a pipeline of open source MATLAB (MathWorks; version R2021a) functions to correct for motion artifacts, segment cells and extract transients. All analyses were conducted in MATLAB. All custom code related to this study are available at https://github.com/akeinath/MorphExperiments.

For manuscripts utilizing custom algorithms or software that are central to the research but not yet described in published literature, software must be made available to editors and reviewers. We strongly encourage code deposition in a community repository (e.g. GitHub). See the Nature Research guidelines for submitting code & software for further information.

Data

Policy information about availability of data

All manuscripts must include a data availability statement. This statement should provide the following information, where applicable:

- Accession codes, unique identifiers, or web links for publicly available datasets
- A list of figures that have associated raw data
- A description of any restrictions on data availability

The complete dataset for all experiments are publicly available on the DRYAD repository at https://doi.org/10.5061/dryad.2z34tmpp9 or via request to the corresponding authors.

Field-specific reporting

Please select the one below that is the best fit for your research. If you are not sure, read the appropriate sections before making your selection.

☒ Life sciences ☐ Behavioural & social sciences ☐ Ecological, evolutionary & environmental sciences

For a reference copy of the document with all sections, see nature.com/documents/nr-reporting-summary-flat.pdf

Life sciences study design

All studies must disclose on these points even when the disclosure is negative.

Sample size	All within-mouse simultaneously-recorded neural populations were large (>100s), set by the number of high-quality putative neural regions of interest within the field of view as extracted by the standard CNMFE preprocessing pipeline as desribed in the Methods. The number of mice recorded (5) and the lower bound of acceptable minimum simultaneously-recorded population size (100) were set to exceed that of prior publications with this paradigm (Wills et al., 2005).
Data exclusions	One animal was recorded but excluded because of poor cell-tracking and imaging quality across days (<30% registration). Analysis of his data revealed results qualitatively similar to the 5 reported mice.
Replication	Because of the large within-mouse population sizes, we are able to conduct and display many of the primary analyses within each of the 5 mice. This gives us confidence in the replicability of these results.
Randomization	The order of the behavioral paradigm was randomized within-sequence and across mice subject to some order constraints as described in the manuscript.
Blinding	As all five animals participated in the same behavioral paradigm, there were no conditions to which blinding would be applicable.

Reporting for specific materials, systems and methods

We require information from authors about some types of materials, experimental systems and methods used in many studies. Here, indicate whether each material, system or method listed is relevant to your study. If you are not sure if a list item applies to your research, read the appropriate section before selecting a response.

Materials & experimental systems

n/a	Involved in the study	
☒	☐	Antibodies
☒	☐	Eukaryotic cell lines
☒	☐	Palaeontology and archaeology
☐	☒	Animals and other organisms
☒	☐	Human research participants
☒	☐	Clinical data
☒	☐	Dual use research of concern

Methods

n/a	Involved in the study	
☒	☐	ChIP-seq
☒	☐	Flow cytometry
☒	☐	MRI-based neuroimaging

Animals and other organisms

Policy information about studies involving animals; ARRIVE guidelines recommended for reporting animal research

Laboratory animals	Naive male mice (C57Bl/6, Charles River). Mice were 6-10 weeks old at the time of their first surgery, and 9-16 weeks old at initiation of the behavioral paradigm.
Wild animals	This study did not include wild animals.
Field-collected samples	This study did not include field-collected samples.
Ethics oversight	All experiments were carried out in accordance with McGill University and Douglas Hospital Research Centre Animal Use and Care Committee (protocol #20157725) and with Canadian Institutes of Health Research guidelines.

Note that full information on the approval of the study protocol must also be provided in the manuscript.
